# Effect of Microwave Pretreatment on the Antioxidant Activity and Stability of Enzymatic Products from Milk Protein

**DOI:** 10.3390/foods11121759

**Published:** 2022-06-15

**Authors:** Xue Yang, Xiaofeng Ren, Haile Ma

**Affiliations:** 1 School of Basic Medical Sciences, Chengde Medical University, Chengde 067000, China; 2 Jiangsu Provincial Key Laboratory for Food Processing, School of Food and Biological Engineering, Jiangsu University, Zhenjiang 212013, China; renxiaofeng@ujs.edu.cn (X.R.); mhl@ujs.edu.cn (H.M.)

**Keywords:** microwave pretreatment, antioxidant activity, stability, enzymatic products, milk protein

## Abstract

The effects of microwave pretreatment on the antioxidant activity and stability of enzymatic products from milk protein (MP) were studied. The peptide content, molecular weight distribution, and amino acid composition of MP hydrolysate were also measured to explain the change of antioxidant activity under microwave pretreatment. The results showed that microwave pretreatment increased the degree of hydrolysis of MP with the power of 400 W for the highest value. The DPPH scavenging activity and the total antioxidant capacity of MP pretreated by microwave with a power of 300 W presented the highest effect and increased by 53.97% and 16.52%, respectively, compared to those of control. In addition, the results of thermal stability and in vitro digestion of MP hydrolysate showed that the MP hydrolysate pretreated by microwave exerted excellent antioxidative stability, especially for the microwave power of 300 W. After pretreated with microwave, the peptide content increased as the rise of power and it reached the peak at the power of 400 W. The molecular weight of MP hydrolysate pretreated by microwave with the power of 300 W showed more percentage of peptides between 200 Da and 500 Da. The result of amino acid composition showed that total amino acid (TAA) content of MP hydrolysate pretreated by microwave with power of 400 W showed the highest value, which increased by 7.58% compared to the control. The ratio of total hydrophobic amino acids to the TAA of MP hydrolysate showed the most increased amplitude with the microwave power of 300 W. The antioxidant activity of MP hydrolysate was related to the peptide content, and it was also relevant to the amino acid category and content. In conclusion, microwave pretreatment is an effective method for the preparation of antioxidant peptides and an increase in antioxidant stability.

## 1. Introduction

Milk is rich in high-quality protein, and it is an important food product to humans because of its nutritional value [1]. Milk protein (MP) contains many active amino acid sequences, and it can hydrolyze under the action of protease to generate peptides with bioactivities [2], such as antihypertensive, antioxidant, immunomodulatory, and antimicrobial activities [3]. The bioactivity of peptides is related to the type of protease [4], the degree of hydrolysis (DH) or enzymatic time [5], the molecular distribution of the peptides [6], and the amino acid composition [7]. The optimization of enzymatic parameters can improve the activity of the product to a certain extent. However, it is still unable to improve the enzymatic reaction efficiency and product activity of protein hydrolysate because of the tight structure of protein material.

In recent years, many new physical processing technologies, such as ultrasound [8,9], ultra-high pressure [10], microwave [11,12], etc., are applied to assist hydrolysis in obtaining a higher content or bioactivity of peptides from foodborne protein. Some of the equipment such as a high hydrostatic pressure and irradiation is very expensive and hence not readily available, especially for home scale use [13]. Compared with other traditional extraction methods, microwave-assisted extraction has the advantages of a rapid and uniform heating, a high thermal efficiency, no pollution, and a high solvent recovery rate, which can significantly shorten the extraction time, improve the extraction rate, and save costs [14]. Microwave refers to electromagnetic waves with a frequency of 300 MHz–300 GHz and wavelengths between 1 mm and 1 m. The basic properties of microwaves usually show three characteristics: penetration, reflection, and absorption. Foodborne protein can absorb microwaves and cause the modification of protein structure. Researches showed that microwave assisted enzymatic hydrolysis could shorten enzymatic time, improve the reaction rate and increase the peptide yield [15,16]. The bioactive peptides are commonly used as antioxidant bioactive products or are added to other foods as a natural antioxidant additive, and most applications of bioactive peptides involve thermal treatment. In addition, some foodborne bioactive peptides fail to show the functional activity after oral administration in vivo due to the excessive degradation in the gastrointestinal tract. These two factors (thermal treatment and oral administration in vivo) significantly influence the bioactivity of protein hydrolysate [17,18]. However, most of the researchers studied the effects of microwave pretreatment on the bioactive activity of protein hydrolysate and little research aimed at the antioxidant activity and antioxidant stability of hydrolysate from the material of MP after a high temperature and gastrointestinal digestion.

Therefore, the objectives of this study were to investigate the effect of microwave pretreatment on the antioxidant activity and stability of enzymatic products from MP. The specific research contents were divided into three related parts: (1) to investigate the effects of microwave pretreatment on the antioxidant activity of MP hydrolysate; (2) to study the stability of MP hydrolysate under high temperatures at different times and digestion in vitro; and (3) to study the effects of microwave pretreatment on the characterizations of MP hydrolysate.

## 2. Materials and Methods

### 2.1. Materials

Pasteurized fresh milk was bought from Bright Dairy Food Co., Ltd. (Shanghai, China) with the protein content of 3.4% and fat content of 0%. Alcalase 2.4 L with an activity of 196,636 U/mL (determined by the Folin–phenol method) was purchased from Novozymes Biotechnology Co. Ltd. (Tianjin, China). 1,1-diphenyl-2-picrylhydrazyl (DPPH) was purchased from Sigma-Aldrich Corp (Saint Louis, MO, USA). Pepsin and pancreatin were bought from Aladdin Bio-Chem Technology Co., Ltd. (Shanghai, China). Aprotinin (6511 Da), bacitracin (1422 Da), Gly-Gly-Tyr-Arg (451.2 Da) and Glycyl-Glycyl-Glycine (189.1 Da) were bought from Beijing Enjiayi Tech Co., Ltd. (Beijing, China). All other reagents used in the experiment were of analytical grade.

### 2.2. Microwave Pretreatment of MP

A 400 mL of fresh milk was added with 4 mol/L NaOH to adjust the pH of the solution to 8.5. Then it was put into microwave equipment (NN-DS59JB, Panasonic Appliances Microwave Oven (Shanghai, China) Co., Ltd.) for pretreatment. Then the microwave power was adjusted to 200, 300, 400, 600, 800, and 1000 W, respectively, with the pretreated time of 2 min. The MP without microwave pretreatment (the power of 0 W) was set as control.

### 2.3. Enzymatic Hydrolysis of MP

The enzymatic hydrolysis of MP was conducted according to our previous study [19]. After microwave pretreatment, the MP was cooled to 50 °C using a water bath. Then the pH of MP was adjusted to 8.5, and the alcalase was added to initiate the enzymatic reaction. During the enzymatic process, the pH of the reaction system was maintained at 8.5 using 1 mol/L NaOH, and the consumption of NaOH was recorded. After 60 min of the enzymatic reaction, the sample was put in boiled water to inactive enzyme. Then it was centrifuged at 12,000 g for 10 min and the supernatant was collected for further analysis.

### 2.4. The Degree of Hydrolysis (DH) of MP

The DH of MP was determined according to the method of Adler-Nissen [20] by the pH-stat method using the following Equation (1):

(1) $$DH\left(\%\right)=\frac{h}{h_{tot}}\times 100=\frac{V\times N}{\alpha \times m\times h_{tot}}\times 100$$

where, *V* is the consumption of NaOH solution (mL); *N*: molar concentration of NaOH solution (mol/L), which is 1 mol/L in this paper; *α* is the average degree of dissociation of α-NH_2_ of MP, which is 0.96 in this paper; *m* is the protein amount of MP (g); *h_tot_* is the number of peptide bonds per unit mass, which is 8.2 mmol/g of MP [21].

### 2.5. Antioxidant Activity Analysis

#### 2.5.1. DPPH Scavenging Capacity Assay

The DPPH assay was done according to the method of Chen et al. [22] with some modifications. A 2 mL of 0.1 mol/L DPPH (dissolved in ethanol) was added to 2 mL hydrolysate. Then the mixture was kept in the dark at 37 °C for 30 min and the absorbance was quickly measured after the reaction at 517 nm by ultraviolet spectrum. The DPPH scavenging activity was calculated by the following Equation (2):

(2) $$DPPH\;scavenging\;activity\;\left(\%\right)=1-\frac{A_{1}-A_{2}}{A_{0}}\times 100$$

where, *A*_0_: the absorbance of blank (ethanol instead of protein-hydrolysate); *A*_1_: the absorbance of sample; *A*_2_: the absorbance of background (ethanol instead of DPPH).

#### 2.5.2. Total Antioxidant Capacity Assay

The procedure followed the method of Thaipong et al. [23] with some modifications [24]. An equal volume of 7.4 mmol/L ABTS and 2.6 mmol/L potassium persulfate was mixed and reacted for 12 h at room temperature in the dark to prepare ABTS working solution. Diluted the working solution with absolute ethanol so that its absorbance at 734 nm was 0.7 ± 0.02 units at 734 nm using the spectrophotometer. The 1 mL of MP hydrolysate was mixed with 4 mL ABTS solution and reacted for 2 h in a dark condition. Then the absorbance was measured at 734 nm using the spectrophotometer. The total antioxidant capacity was calculated by the following Equation (3):

(3) $$Total\;antioxidant\;capacity\;\left(\%\right)=1-\frac{A_{4}-A_{5}}{A_{3}}\times 100$$

where, *A*_3_: the absorbance of the sample replaced by distilled water; *A*_4_: the absorbance of the sample; *A*_5_: the absorbance of the ABTS working solution replaced by distilled water.

### 2.6. Stability of Antioxidant Activity of MP Hydrolysate

#### 2.6.1. Effects of Heat Treatment at different times on Antioxidant Activity of MP Hydrolysate

The heat stability of MP hydrolysate at different times was conducted according to the method of Escudero et al. [25] with some modifications. The MP hydrolysates were treated at 50, 75, 90, 105, and 120 °C, respectively, for 10 min. Furthermore, the hydrolysates were treated at 105 °C for 5, 10, 15, 30, and 60 min. Then the samples were cooled to 25 °C and centrifuged at 12,000× *g* for 10 min. The supernatant was collected to determine the antioxidant activity.

#### 2.6.2. Antioxidant Activity of MP Hydrolysate after Digestion In Vitro

The antioxidant activity of MP hydrolysate after digestion by gastric proteases was assessed using pepsin and pancreatin according to the method of Laparra [26] with some modifications [27]. After adjusting the pH to 2.0 with 4 mol/L HCl, the MP hydrolysate was added with 2% (enzyme to substrate ratio) pepsin. The digestion was kept at 37 °C for 2 h under continuous stirring. Then the pH of the digestion solution was adjusted to 7.5 with 4 mol/L NaOH and 2% (enzyme to substrate ratio) pancreatin was added. After digestion at 37 °C for 3 h, the enzyme was inactivated by heating in boiled water for 10 min. Then the sample was centrifuged at 12,000× *g* for 10 min and the supernatant was used for the analysis of antioxidant activity.

### 2.7. Peptide Content

A 15% of TCA (*w/v*) was added to MP hydrolysate with a volume ratio of 1:2. The mixture was reacted at 25 °C for 1 h to precipitate macromolecular proteins. Then the sample was centrifuged at 12,000× *g* for 10 min and the supernatant was used to determine the peptide content according to the Folin–phenol method [28].

### 2.8. Molecular Weight Distribution of MP Hydrolysate

The molecular weight distribution of MP hydrolysate was determined by high performance liquid chromatography. Chromatographic conditions were as follows: column of TSK gel G2000 SWXL 300 mm × 7.8 mm, flow velocity of 0.5 mL/min, injection volume of 10 μL, and column temperature of 30 °C. The standard solutions with 0.1% mass concentration were prepared with the mobile phase. After filtering with a 0.45 μm water membrane, the standard solutions were injected, respectively, to obtain the chromatograms of a series of standard products. The calibration curve was obtained by plotting the logarithm of the relative molecular mass (lgMr) against the retention time. The MP hydrolysates were also injected after filtering with 0.45 μm water film. Substitute the sample chromatogram data into the calibration curve for integration to obtain the corresponding percentage of the peak area.

### 2.9. Amino Acid Analysis

The amino acid composition of MP hydrolysate was analyzed by an automatic amino acid analyzer (S433D, Sykam, Eresing, Germany) [29]. A 6 mol/L HCl was added to MP hydrolysate and the mixture was sealed in a tube at 110 °C for 24 h. Then the sample was centrifuged at 12,000× *g* for 10 min. The supernatant was collected and filtered by 0.22 μm water film. Then it was injected into automatic amino acid analyzer.

### 2.10. Statistical Analysis

Statistical analysis was performed with SPSS 23.0. One-way ANOVA analysis and LSD multiple range tests were performed. The mean and standard deviation were reported and the significant differences among mean values were at *p* < 0.05. Experiments for each sample were done in triplicate.

## 3. Results

### 3.1. The DH of MP

**Figure 1:**
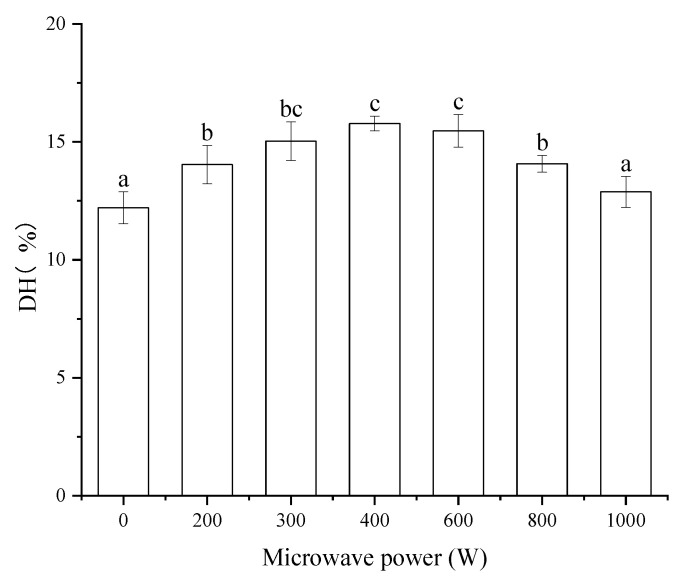
The effects of microwave pretreatment on the DH of MP. Results represent the means of three determinations ± standard deviation. Means with different superscripts (a, b, c) are significantly different (*p* < 0.05).

The DH of protein has significant correlations to the bioactivity of protein-hydrolysates. The effect of microwave pretreatment on the DH of MP was shown in Figure 1. All the microwave pretreatments increased the DH of MP except for the microwave power of 1000 W. Compared to the control (the microwave power of 0 W), the MP pretreated by microwave with 400 W showed the highest DH with the value of 15.78%, which increased by 29.28%. The DH of MP pretreated by microwave with the power of 300 W and 600 W had no significant difference (*p* > 0.05) compared to that of 400 W. The DH values reported in this study are similar to that of MP pretreated by microwave [30]. The DH of MP increase might be due to the protein unfolding associated with the pretreatment [31]. Microwaves may promote the cross-linking of protein molecules, especially the disulfide bonds and destroy the covalent or non-covalent bonds between protein molecules [32]. The microwave pretreatment made the hydrophobic groups of protein exposed. As the microwave power went up, the protein molecular started to aggregate. When the microwave power increased to 1000 W, the aggregation degree of protein was deeper [33]. Proper protein modification favors proteolysis, while excess denaturation has the opposite effect. This resulted in the reduction of the DH of MP at a microwave power of 1000 W compared to others.

### 3.2. Antioxidant Activity of MP Hydrolysate

**Figure 2:**
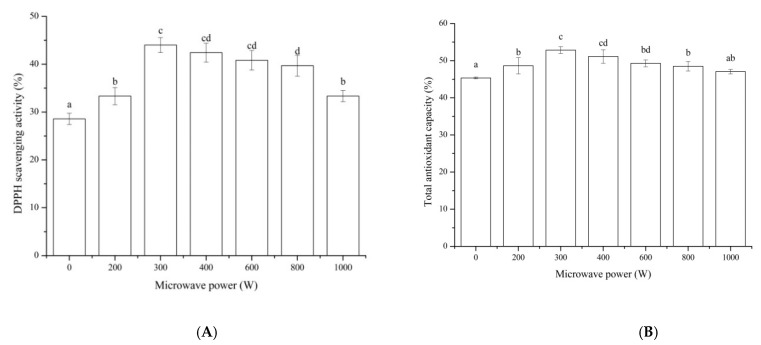
Antioxidant activity: DPPH scavenging activity (**A**) and total antioxidant capacity (**B**) of MP hydrolysate after pretreated by microwave. Results represent the means of three determinations ± standard deviation. Means with different superscripts (a, b, c, d) are significantly different (*p* < 0.05).

DPPH scavenging activity is a commonly used index in many studies for evaluating the free radical scavenging potentials of natural compounds in vitro and their antioxidant effect [34]. The impacts of microwave pretreatment on the DPPH scavenging activity of MP hydrolysate were shown in Figure 2A. All the microwave pretreatments of MP with different powers showed a higher DPPH scavenging activity compared to that of the control. The DPPH scavenging activity of the microwave with the power of 300 W showed the highest value of 43.99% and increased by 53.97% compared to that of the control, and it had no significant influence compared to that of 400 W and 600 W. The total antioxidant capacity of MP hydrolysate after pretreated by microwave (Figure 2B) showed the similar trend to that of the DPPH scavenging activity. The total antioxidant capacity of MP pretreated by microwave with the power of 300 W showed the highest value of 52.83%, which increased by 16.52% compared to that of the control. These results were in line with that of the DH of MP (Figure 1). Research showed that high values of DH are positively correlated with a high bioactivity [35]. In addition, they were inconsistent with that of sunflower meal protein, which showed that the microwave power of 400 W was higher than that of 300 W and 600 W [36]. The difference might be relevant in the solubility of protein material. The material of MP was soluble, and it could be affected at a lower microwave power compared to insoluble protein.

### 3.3. Stability of MPHydrolysate

**Figure 3:**
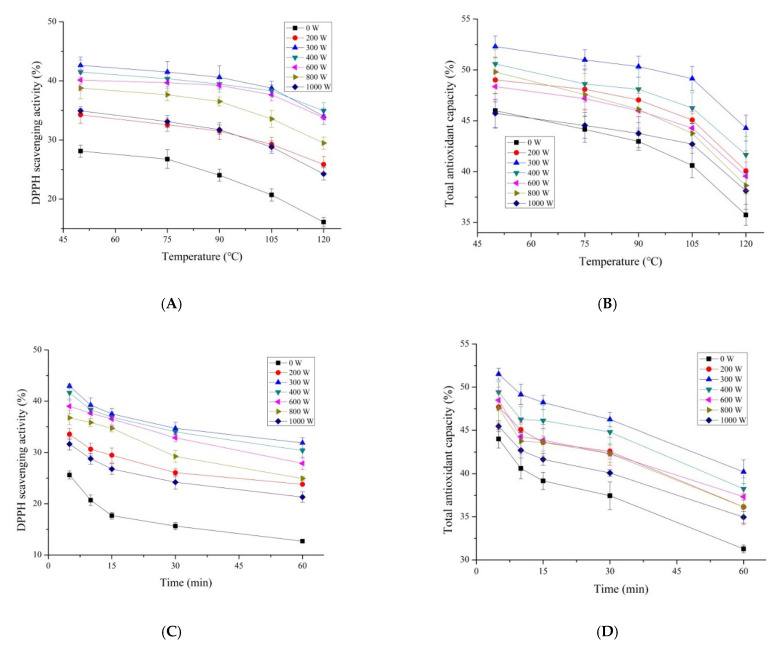
Thermal treatment on the DPPH scavenging activity (**A**), total antioxidant capacity (**B**), treatment time on the DPPH scavenging activity (**C**), and total antioxidant capacity (**D**) of MP hydrolysate after pretreated by microwave. Results represent the means of three determinations ± standard deviation.

The bioactive peptides were used for antioxidant bioactive products or added to other foods as a natural antioxidant. Therefore, it is necessary to evaluate their stability under high temperature processing conditions. The effects of temperature on the antioxidant activity of MP hydrolysate after being pretreated by microwave are listed in Figure 3. The results showed that microwave pretreatment could increase the heat stability of MP hydrolysate compared to that of the control. All the pretreatments decreased the magnitude of the reduction in antioxidant activity. The DPPH scavenging activity of MP hydrolysate pretreated by microwave showed a higher stability below the temperature of 90 °C. Compared to the control, the MP hydrolysate with the pretreatment of 300 W microwave power showed the highest DPPH scavenging activity with the value of 34.01% and increased by 89.89% (Figure 3A) at the temperature of 120 °C. The total antioxidant capacity of MP hydrolysate decreased as the temperature went up (Figure 3B). The MP hydrolysate pretreated by microwave with the power of 300 W showed the highest activity with the value of 44.28%. Similar results were observed on the effect of heat processing time on the antioxidant activity of MP hydrolysate pretreated by microwave (Figure 3C,D). Microwave pretreatment increased the antioxidant stability of MP hydrolysate at a high temperature from 5 to 60 min compared to that of the control. The MP pretreated by microwave with the power of 300 W presented the highest antioxidant stability. The stability rate of the DPPH scavenging activity and total antioxidant capacity of MP hydrolysate pretreated by microwave with the power of 300 W increased by 21.19% and 8.02%, respectively. The MP hydrolysate pretreated by microwave had a certain anti-oxidative stability, especially for the DPPH scavenging activity. This indicated that a high temperature (above 90 °C) could lead to the loss of antioxidant activity of peptides from MP. Microwave pretreatment reduced this loss. These results were consistent with that of Zhao [37] who found that the antioxidant activity of protein hydrolysate from Spanish Mackerel decreased when treated under 80 and 100 °C. And the same phenomenon was found in Antarctic krill (*Euphausia superba*) proteins [38].

### 3.4. In Vitro Digestion of MP Hydrolysate

**Figure 4:**
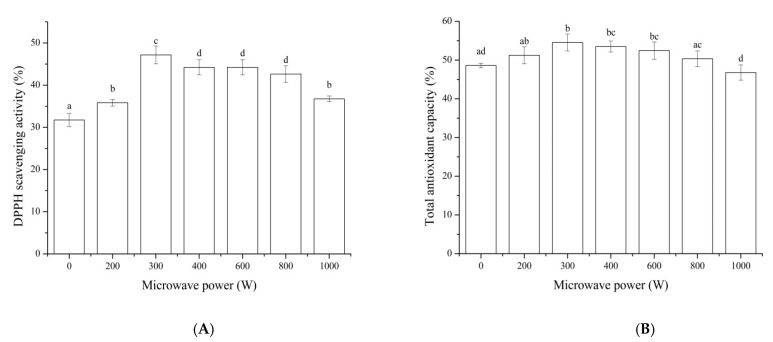
The effect of digestion in vitro on DPPH scavenging activity (**A**) and total antioxidant capacity (**B**) of MP hydrolysate after pretreated by microwave. Results represent the means of three determinations ± standard deviation. Means with different superscripts (a, b, c, d) are significantly different (*p* < 0.05).

Some food-protein-derived bioactive peptides failed to show functional activity after oral administration in vivo due to the fact that they are hydrolyzed in the gastrointestinal tract to peptides with a reduced activity [39]. Therefore, the antioxidant activity of MP hydrolysate digested in vitro was evaluated. As shown in Figure 4, the DPPH scavenging activity and total antioxidant capacity of MP hydrolysate after being pretreated by microwave increased with the power of 300 W of the highest value. This result indicated that peptides in MP hydrolysate could make use of the gastrointestinal digestive enzymes to generate more antioxidant peptides. These results were inconsistent with the literatures [40,41], which showed the influence of resistance to digestion or the breaking down of peptides in the gastrointestinal tract. The difference might result from the difference in protein material, type of protease, and enzymatic parameter.

### 3.5. Peptide Content

**Figure 5:**
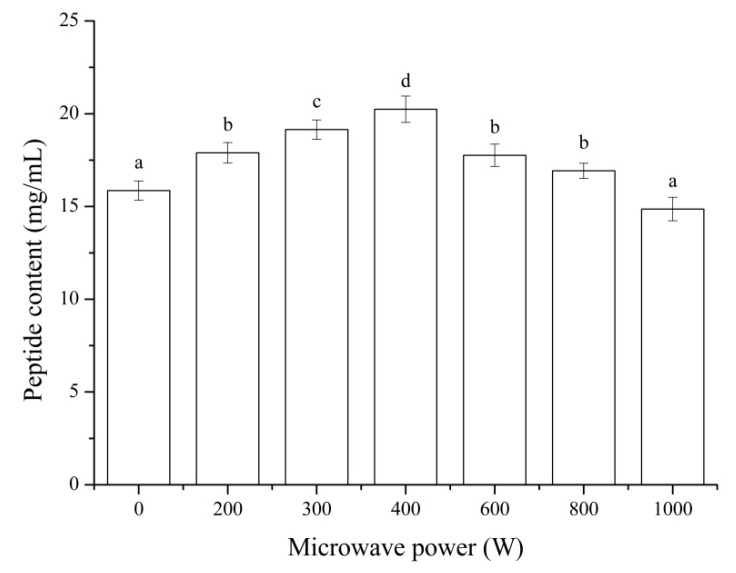
Peptide content of MP hydrolysate after pretreated by microwave. Results represent the means of three determinations ± standard deviation. Means with different superscripts (a, b, c, d) are significantly different (*p* < 0.05).

The effects of microwave pretreatment with different powers on the peptide content were shown in Figure 5. The peptide content of MP hydrolysate after pretreated by microwave increased significantly (*p* < 0.05) compared to that of the control (without microwave pretreatment) except for the microwave power of 1000 W. The highest peptide content was observed at a microwave power of 400 W with the value of 20.24 mg/mL and increased by 27.66% compared to the control. Microwave pretreatment was capable of enhancing the solubilization and protein recovery in subsequent enzymatic hydrolysis. The peptide content was a positive correlation with the DH of MP (Figure 1). These results were similar to that of microwave pretreatment on the chicken feather [42] and defatted soybean meal [43].

### 3.6. Molecular Weight Distribution of MP Hydrolysate

**Figure 6:**
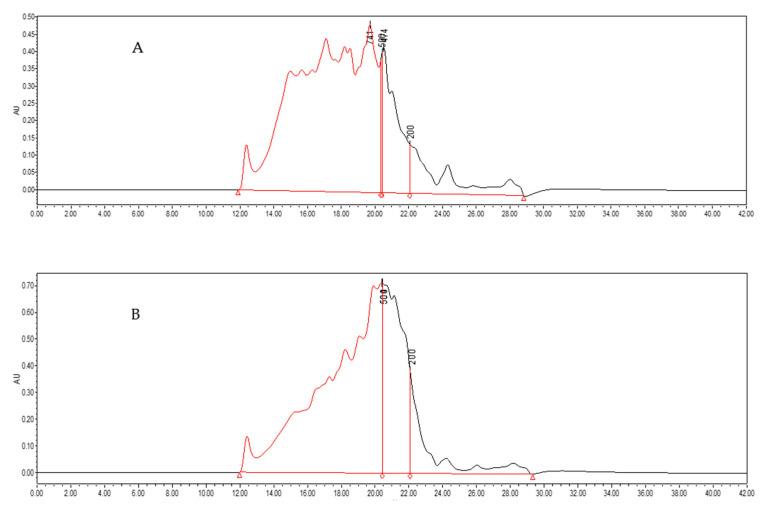
Chromatogram of molecular weight distribution of hydrolysate from MP. (**A**) Control (without the microwave power of 0 W) and (**B**) microwave pretreatment of MP with the power of 300 W.

The effect of microwave pretreatment with the power of 300 W on the molecular weight distribution chromatogram of MP hydrolysate was shown in Figure 6. From the chromatogram, it could be seen that after microwave pretreatment, MP with a larger molecular weight was hydrolyzed compared to that of the control. As shown in Table 1, the peak area of the MP hydrolysate pretreated by microwave was higher under the same injection volume. This was related to the protein/peptide content of hydrolysate, and it was consistent with the results of the peptide content (Figure 5). The molecular weight of MP hydrolysate between 200 Da and 500 Da was much higher than that of the control and the molecular weight more than 500 Da decreased with more protein/peptide hydrolyzed to short chain peptides. The results showed that short chain peptides had a high contribution rate for bioactivity antioxidant [44,45]. These results indicated that microwave pretreatment could increase the DH of MP, the peptide content of MP hydrolysate, and the content of small molecular peptides, which improved the antioxidant activity of MP hydrolysate. This proved that microwave pretreatment of MP was an effective way to promote the enzymatic process.

### 3.7. Amino Acid Composition

The results of the amino acid content of MP hydrolysate pretreated by different microwave powers are listed in Table 2. All the hydrolysates were rich in Asp, Glu, Met, Leu, and Lys. The total amino acid (TAA) content of MP hydrolysate pretreated by microwave with the power of 400 W showed the highest value, which increased by 8.03% compared to the control. This result is consistent with that of the peptide content (Figure 5). The ratio of total hydrophobic amino acids (THAA) to the TAA of MP hydrolysate showed the highest amplitude with the microwave power of 300 W. The ratio of acidic amino acids (AAA) to basic amino acids (BAA) in MP hydrolysate pretreated by microwave was calculated and that of 200 W showed the highest value. Peptides derived from natural proteins with a higher hydrophobicity have demonstrated excellent antioxidative properties in vitro [46], and the ratio of AAA to BAA might have a better digestive stability during the digestion process [47]. This resulted in the stability of MP hydrolysate after microwave pretreatment (Figure 3 and Figure 4). The peptide content of MP hydrolysate pretreated by microwave with the power of 400 W showed the highest value. However, the antioxidant activity of it was lower than that of 300 W. The result of amino acid analysis explained this phenomenon. The antioxidant activity of MP hydrolysate was related to the peptide content, and it was also relevant to the amino acid category and content.

## 4. Discussion

Most of the research focused on the effects of microwave pretreatment on the bioactive activity of protein hydrolysate, but little research focused on the antioxidant activity and its stability of MP hydrolysate after high temperature and gastrointestinal digestion. Therefore, the objectives of this study were to investigate the effect of microwave pretreatment on the antioxidant activity and stability of enzymatic products from milk protein. The microwave pretreatment increased the DH of MP and antioxidant activity of MP hydrolysate except for 1000 W. This might be relevant to the exposure of hydrophobic groups of protein during microwave process and as the microwave power went up, the protein molecular start to aggregate, which is not beneficial for enzymatic hydrolysis [33]. Proper protein modification favors proteolysis, while excess denaturation has the opposite effect. Our previous research showed that a high temperature (95 °C, 20 min) caused the excessive aggregation of protein molecules of grass carp, which is not conducive to proteolysis [9]. This resulted in the reduction of the DH of MP at a microwave power of 1000 W compared to others. The microwave power of 400 W showed the highest DH, but it had no significant difference (*p* > 0.05) compared to that of 300 W.

The antioxidant activity of MP hydrolysate pretreated by microwave with the power of 300 W showed the highest value, and it had a different influence (*p* < 0.05) compared to microwave pretreatment with the power of 400 W. In addition, the antioxidant activity of MP hydrolysate pretreated by microwave with a power of 300 W during thermal treatment and digestion in vitro showed better stability compared to others. This result was inconsistent with that of the DH of MP. It might be relevant to the peptide content or amino acid composition of MP hydrolysate after different pretreatments. Then, the characterization of MP hydrolysate was researched to reveal the reason why different microwave powers presented different results in antioxidant activity and its stability. From the result it could be seen that the peptide content of MP hydrolysate was consistent with that of the DH of MP. The molecular weight of MP hydrolysate pretreated by microwave showed a higher content of peptide between 200 Da and 500 Da, and a molecular weight more than 500 Da decreased significantly. This showed that microwave pretreatment could reduce the amount of macromolecular protein and increase the uniformity of MP hydrolysate. The result of amino acid analysis showed that the total amino acid content of MP hydrolysate after being pretreated by a microwave power of 400 W showed the highest value. This result was in line with that of peptide content and DH. The ratio of THAA to TAA of MP hydrolysate showed the highest amplitude with the microwave power of 300 W, and this result was consistent with that of the antioxidant activity. From the results it could be seen that the microwave pretreatment of MP could increase the antioxidant activity and its stability of MP hydrolysate during high temperature and digestion in vitro, and the antioxidant activity was not only related to the peptide content. What is more, it was relevant to the amino acid category and content in MP hydrolysate.

It is hoped that this research can be helpful in the preparation and application of antioxidant peptides from MP. It is also hoped that the microwave pretreatment method can provide an effective method for the preparation of relatively stable antioxidant peptides from MP. The milk antioxidant peptides pretreated by microwave has broad application prospects in the prevention and treatment of free radical-induced diseases and anti-aging. Meanwhile, the further studies are focused on the design of microwave equipment with more choice among 300 W and 400 W, for example, 320, 340, 360, and 380 W to choose a better microwave power for the preparation of active peptides. Other thermophysical processing methods can also be discussed on the preparation of antioxidant peptide from MP.

## 5. Conclusions

All the microwave pretreatments increased the DH of MP except for the power of 1000 W and that with 400 W showed the highest value compared to the control. All the microwave pretreatments of MP showed a higher DPPH scavenging activity and total antioxidant capacity with 300 W as the highest value and increased by 53.97% and 16.52%, respectively, compared to that of the control. The DPPH scavenging activity of MP hydrolysate pretreated by microwave showed a higher stability below the temperature of 90 °C. After digestion in vitro, the antioxidant activity of MP hydrolysate increased, and this result indicated that the gastrointestinal digestive enzymes could be enzymatically decomposed into antioxidant peptides. The peptide content of MP hydrolysate after pretreated by microwave increased significantly (*p* < 0.05) except for the 1000 W microwave power. The highest peptide content was observed at a microwave power of 400 W, which was consistent with the result of the DH of MP. The result of molecular weight distribution showed that the molecular weight between 200 Da and 500 Da of MP hydrolysate pretreated by microwave with power of 300 W was much higher than that of the control. The TAA content of MP hydrolysate pretreated by microwave with a power of 400 W showed the highest value, which increased by 7.58% compared to the control. This result is consistent with that of peptide content and the DH of MP. The ratio of THAA to TAA of MP hydrolysate showed the highest amplitude with the microwave power of 300 W. The results showed that after microwave pretreatment, the peptide content, the ratio of small molecule peptides, and the antioxidant peptide content increased, which resulted in the increase in antioxidant activity and stability. In conclusion, microwave pretreatment is an effective method for the preparation of antioxidant peptides and increased the antioxidant stability.

## Figures and Tables

**Table 1:** The molecular weight distribution of hydrolysate from MP.

Molecular Weight (Da)	Control		Microwave Pretreatment	
	Peak Area 10^7^ (mV × s)	Content (%)	Peak Area 10^7^ (mV × s)	Content (%)
>500	15.08	77.75	15.13	64.53
200–500	2.51	12.94	5.98	25.52
<200	1.81	9.31	2.33	9.95
Total	19.40	100.00	23.44	100.00

Microwave pretreatment with the power of 300 W.

**Table 2:** Amino acid content of MP hydrolysate pretreated by microwave (mg).

Category	0	200	300	400	600	800	1000
ASP	1.12	1.22	1.38	1.25	1.28	1.21	1.20
THR	0.38	0.37	0.42	0.38	0.41	0.41	0.35
SER	1.12	1.14	0.94	1.14	0.92	1.15	0.92
GLU	1.71	2.25	2.27	2.32	2.00	1.84	2.21
GLY	0.46	0.50	0.49	0.48	0.46	0.48	0.43
ALA	0.41	0.48	0.46	0.43	0.42	0.42	0.37
CYS	0.01	0.07	0.02	0.00	0.02	0.01	0.03
VAL	0.49	0.47	0.59	0.50	0.56	0.54	0.51
MET	1.39	1.42	1.47	1.46	1.44	1.41	1.51
ILE	0.79	0.81	0.86	0.80	0.89	0.85	0.79
LEU	1.36	1.37	1.49	1.39	1.44	1.43	1.38
TYR	0.82	0.80	0.89	0.82	0.88	0.85	0.81
PHE	0.40	0.45	0.53	0.51	0.48	0.44	0.47
HIS	0.68	0.56	0.63	0.55	0.66	0.75	0.55
LYS	3.62	3.24	3.34	3.83	3.79	3.71	3.19
ARG	0.88	0.88	0.98	0.98	0.99	0.94	0.93
PRO	0.47	0.54	0.61	0.56	0.56	0.54	0.55
AAA *	2.84	3.46	3.65	3.58	3.28	3.05	3.41
BAA **	5.18	4.68	4.94	5.36	5.44	5.40	4.67
THAA ***	5.31	5.53	6.00	5.65	5.78	5.64	5.58
TAA	16.12	16.53	17.35	17.42	17.18	16.99	16.19

* THAA, total hydrophobic amino acids, involves Met, Phe, Val, Leu, Ile, Pro, and Ala. ** AAA represents acidic amino acids (Asp and Glu). *** BAA represents basic amino acids (Arg, Lys, and His).
